# Adolescents’ health literacy and health-related quality of life: The mediating role of self-efficacy

**DOI:** 10.1016/j.puhip.2026.100849

**Published:** 2026-08-31

**Authors:** Kristin Sofie Waldum-Grevbo, Solveig Holen, Tore Wentzel-Larsen, Åse Sagatun

**Affiliations:** a Centre for Child and Adolescent Mental Health, Eastern and Southern (RBUP)/PILAR, Oslo, Norway; b VID Specialized University (VID), Oslo, Norway

**Keywords:** Health literacy, Self-efficacy, Health-related quality of life, Adolescence, Sex, Public health, School, School health

## Abstract

**Objectives:**

Enhancing Health-related quality of life (HRQoL) among adolescents is a major public health goal. HRQoL is associated with the modifiable factors health literacy and self-efficacy. Theory and research suggest that individuals with higher health literacy are more capable of managing health experiences, which may strengthen self-efficacy and improve HRQoL. However, the mediating role of self-efficacy in the association between health literacy and HRQoL is scarcely studied. The overall aim of this study is to examine the relationship between self-reported health literacy and HRQoL, and the potential mediating role of self-efficacy in 13-year-old boys and girls.

**Study design:**

The study employed a cross-sectional, school-based design.

**Methods:**

Self-report data were collected from 1425 13-year-old students (71% response rate) across 48 schools in 19 municipalities across Norway. HRQoL, health literacy, and self-efficacy were assessed using KIDSCREEN-27, HLSAC, and GSE-5, respectively. Mediation analysis was conducted within a causal mediation framework to estimate the potential total, direct, and indirect effects on HRQoL associated with a hypothetical 10-percentage-point increase in the health literacy score, with general self-efficacy as the proposed mediator.

**Results:**

Overall, higher health literacy showed a significant increase in HRQoL across all subscales.

The largest total effects were observed for the School Environment domain. A hypothetical 10 percentage-point increase in health literacy was associated with increases of 2.47 T-score points (95% CI 2.08-2.85) among girls and 1.86 (95% CI 1.40-2.35) among boys. The proportion potentially mediated through self-efficacy ranged from 30% to 76% across HRQoL subscales.

**Conclusions:**

This study contributes to the literature by suggesting that general self-efficacy may be a potential mediator of the association between health literacy and HRQoL. Future interventions targeting health literacy should be evaluated using longitudinal designs that can assess the temporal ordering from health literacy to general self-efficacy and subsequently to HRQoL.

## 1 Introduction

Adolescence is a significant period of transition, with rapid physical, emotional, cognitive, and social development. [1] This phase represents an important opportunity for preventive and health-promoting interventions aimed at supporting healthy development and well-being into adulthood. Health-related quality of life (HRQoL) has emerged as an important determinant of health, as highlighted in the United Nations’ Sustainable Development Goal 3. [2] HRQoL captures the impact of health on overall well-being, encompassing physical, psychological, and social functioning. [3] Consequently, enhancing HRQoL is a primary overarching aim of public health.

To enhance HRQoL, it is essential to identify modifiable factors that can improve individual well-being and development. According to the World Health Organization, health literacy is a key and potentially modifiable resource in this effort. [4] In Norway, health literacy is defined in line with the Organization for Economic Co-operation and Development (OECD) as an individual's knowledge, motivation, and skills to access, understand, evaluate, and apply health information. [5] Accordingly, Paakkari et al. operationalized health literacy into five dimensions integrated into school learning outcomes: theoretical knowledge, practical knowledge, critical thinking, self-awareness, and citizenship. [6]

High levels of health literacy in adolescence are linked to beneficial health behaviors such as increased physical activity, a healthy diet, and proper sleep. [7,8] A comparative study on adolescents' health literacy in Europe reveals that levels of health literacy vary by 13.5 percentage points among participating countries. [9] Research studying health literacy and health promotion in school-aged adolescents indicates a positive association between health literacy and HRQoL. [7] Enhancing health literacy may help increase autonomy and personal mastery, as a component of an individual's development towards a better quality of life. [10]

Alongside health literacy, self-efficacy plays an important role in effectively applying knowledge in meaningful ways. [11,12] Self-efficacy originates from social-cognitive theory and refers to individuals' beliefs in their ability to manage and cope with life's challenges. [13] General self-efficacy represents a more stable and optimistic belief in one's capacity to handle everyday demands and adapt to stressful events. [14] Previous research, mainly among adult patient populations, has demonstrated positive associations between health literacy and self-efficacy, suggesting that individuals who better understand their health feel more capable of managing it. [15] Mastery experiences are considered a key source of general self-efficacy, [16] supporting the theoretical assumption that individuals who understand their health conditions feel more confident in managing them, potentially improving HRQoL. [11]

Like health literacy, self-efficacy has been associated with favorable health behaviors and outcomes, including healthier diets, smoking cessation, physical activity, and improved health and quality of life. [17,18] Self-efficacy has been identified as a mediator of the associations of pain, stress and sleep, with HRQoL among adolescents. [[18], [19], [20]] A similar mediating role has been observed in the association between health literacy and quality of life among adults with chronic diseases. [21] Taken together these findings suggest that self-efficacy also mediates in the relationship between health literacy and quality of life among adolescents.

Whether these associations operate similarly for boys and girls remains unclear. Previous research has documented sex differences in self-rated health, with boys generally reporting better HRQoL, and self-efficacy, than girls. [1,22] Although boys and girls generally report confidence in their ability to solve problems, boys tend to report higher levels of confidence. [1] In contrast, a recent European comparative study found no significant sex differences in adolescents’ health literacy. [9] These mixed findings underscore the importance of examining the associations between health literacy, self-efficacy, and HRQoL separately for boys and girls.

Several studies have reported positive associations between health literacy, self-efficacy, and HRQoL and identified self-efficacy as a mediator in related health outcomes. [7,15,[18], [19], [20], [21]] However the potential mediating role of self-efficacy in the association between health literacy and HRQoL among adolescents remains largely unexplored. Theoretically, higher health literacy may enable individuals to better understand and manage health-related experiences, thereby strengthening self-efficacy and positively influencing HRQoL. [[10], [11], [12], [13]]

Based on theory and previous research, we hypothesized that higher health literacy would be associated with higher HRQoL and that this association would be partly mediated by self-efficacy. Accordingly, the aim of this study was to examine the association between self-reported health literacy and HRQoL among 13-year-old and to investigate self-efficacy as a potential mediator. Given established sex differences in self-rated health, potential sex differences were also examined.

## 2 Methods

### 2.1 Study design and population

The present cross-sectional study is based on baseline data from students participating in an intervention study called GuideMe. [23] Data were collected from 48 lower secondary schools in19 municipalities across Southeast and Central Norway, representing both urban and rural areas, with populations ranging from about 1000 to 700,000 citizens. 13-year-old students were recruited in two waves – 2022/23 (wave 1) and 2023/24 (wave 2) – and were followed for one school year.

To enhance representativeness within schools, entire eighth-grade classes were invited to participate. Each school recruited between one and four parallel classes, depending on school size and available resources. Inclusion criteria were enrollment in a participating class and parental consent. Exclusion criteria included intellectual disability or language difficulties that prevented completion of the questionnaires, as well as long-term school absenteeism.

Written parental consent was obtained due to participants’ age, while student consent was provided through questionnaire participation. Both students and parents received standardized information emphasizing voluntary participation and the right to withdraw at any time.

**Fig. 1:**
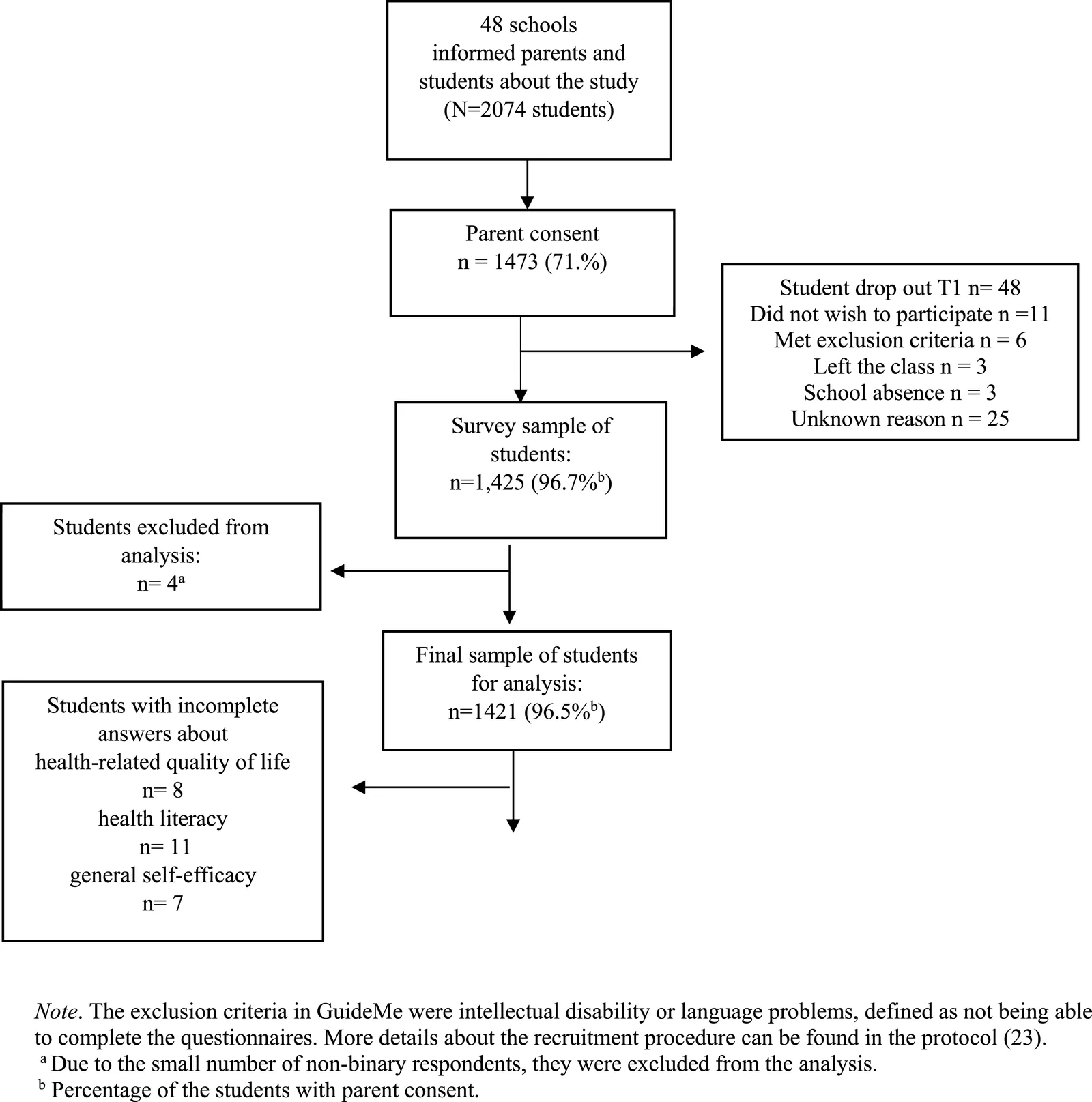
Flowchart of Participants *Note*. The exclusion criteria in GuideMe were intellectual disability or language problems, defined as not being able to complete the questionnaires. More details about the recruitment procedure can be found in the protocol. [23] ^a^ Due to the small number of non-binary respondents, they were excluded from the analysis. ^b^ Percentage of the students with parent consent.

At the start of the semester, the students completed an electronic survey in the classroom. A total of 1425 students (71% of those invited) responded to the survey (Fig. 1), with a sex distribution of 50.3% girls and 49.7% boys. Most participants reported Norwegian as their home language (85%), lived with both parents (70%), and perceived their family's financial situation as similar to that of others (67%).

### 2.2 Measures

#### 2.2.1 Health-related quality of life

HRQoL was measured using the Norwegian version of the self-report instrument KIDSCREEN-27. [24] The instrument was developed for children and adolescents aged 8–18 years and is widely used in public health research. [25] KIDSCREEN-27 includes five subscales: Physical Well-being (5 items), Psychological Well-being (7 items), Autonomy and Parent Relations (7 items), Peer and Social Support (4 items), School Environment (4 items). Items are rated on 5-point Likert scales. Subscale scores were transformed into T-scores (mean = 50, SD = 10) using KIDSCREEN syntax. [a, b, [26]] The instrument has demonstrated good psychometric properties internationally. [25] In the present study, Cronbach's alpha ranged from 0.81 to 0.88 across subscales, reflecting satisfactory reliability.

#### 2.2.2 Health literacy

Health literacy was assessed using the Norwegian version of the Health Literacy Among School-Aged Children (HLSAC). [6] The instrument is commonly used for assessing health literacy among European adolescents in schools. [27] HLSAC is a 10-item self-report scale developed for use among European adolescents. Health literacy is operationalized across five dimensions: theoretical knowledge, practical knowledge, critical thinking, self-awareness, and citizenship, with two items per dimension. Items are rated on a 4-point Likert scale, with a total sum score ranging from 10 to 40. The instrument has shown high internal consistency across countries. [28] In the current study, Cronbach's alpha was 0.90, indicating excellent reliability.

#### 2.2.3 Self-efficacy

Self-efficacy was measured by the Norwegian 5-item version of the General Self-Efficacy Scale (GSE-5). [29] The short form was developed for large population-based studies and is structured as a one-factor scale. Items are rated on a 4-point Likert scale, with sum scores ranging from 5 to 20. The Norwegian version has demonstrated good psychometric properties among adolescents. [16] The Cronbach's alpha in the current study was 0.81, reflecting high internal consistency.

### 2.3 Statistical analysis

HRQoL, health literacy and self-efficacy were studied using descriptive analysis stratified by sex, while sex differences were examined using t-tests.

**Fig. 2:**
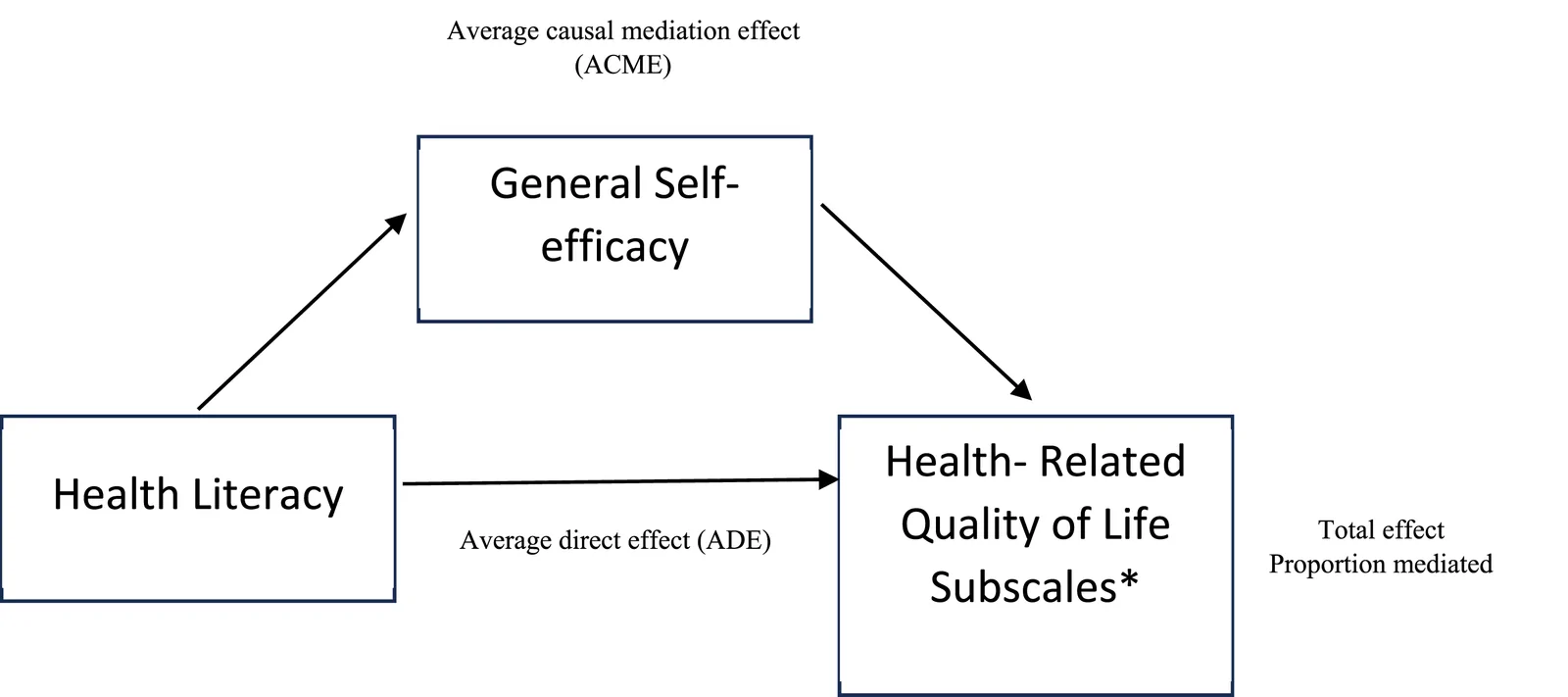
Theoretical mediation model The figure illustrates the hypothesized theoretical mediation pathway between health literacy, general self-efficacy, and HRQoL. Effect estimates for each HRQoL subscale by sex are reported in Table 2. * HRQoL subscales: Physical Well-being, Psychological Well-being, Autonomy and Parent Relations, Peer and Social Support, School Environment.

Health literacy and self-efficacy are conceptually related. To assess potential overlap between the HLSAC and GSE-5 instruments, item-level associations were examined using Spearman's rank correlation coefficients. [30] Mediation analysis was conducted within a causal mediation framework [31] to examine the hypothesized theoretical mediation pathway between health literacy, self-efficacy, and HRQoL (Fig. 2). Interpretation of the estimated direct and indirect effects relies on several assumptions, including the absence of unmeasured confounding in the exposure-outcome, exposure-mediator, and mediator-outcome relationships, as well as correct temporal ordering of the variables. Given the cross-sectional design, these assumptions cannot be fully verified, and the findings should therefore be interpreted with caution. Sensitivity analyses were performed to evaluate the extent to which unmeasured confounding would need to influence the mediator-outcome relationship for the estimated direct and mediated effects to be reduced or change direction.

The potential direct and indirect effects of health literacy on the KIDSCREEN subscales scores: Physical Well-being, Psychological Well-being, Autonomy and Parent Relations, Peer and Social Support and School Environment were estimated (Table 2). The indirect effects represent the potential effects operating through self-efficacy, whereas the direct effects represent the potential effects not mediated by self-efficacy. To improve interpretability, health literacy and self-efficacy, sum scores were linearly transformed to a 0–100 scale.

Separate analyses were conducted for the different KIDSCREEN subscales. Potential direct and indirect effects were estimated for a hypothetical 10 percentage point increase in transformed health literacy score. A difference of about 10 points on a 0–100 scale is commonly considered noticeable. [30] To ensure relevance, potential effects were estimated within the main range of the distributions, avoiding the tails. These intervals corresponded to the 50th–75th percentiles, equivalent to values between 60 and 70 on the transformed scale. KIDSCREEN outcomes were expressed as T-scores. All mediation analyses were stratified by sex and based on complete cases.

Mediation was conducted using the R package *mediation.* [31] The package does not provide a functionality to account for clustered data structures. Clustering effects are generally considered negligible when intra-class correlation coefficients (ICCs) are below 0.05. [32] In the present study the ICCs for the outcome variables ranged from 0.003 to 0.03, indicating minimal clustering at the school level.

The significance level was set to p < 0.05. Descriptive statistics were analyzed using IBM SPSS Statistics version 27.

## 3 Results

### 3.1 Health literacy, self-efficacy and HRQoL scores

Boys scored significantly higher than girls on the measures of health literacy, self-efficacy, and HRQoL (Table 1). Across the HRQoL subscales, boys' mean T-scores ranged from 48.98 to 52.33, whereas girls' mean T-scores ranged from 43.99 to 49.77 (Table 1). Spearman's rank correlation coefficients ranged from 0.14 to 0.35, indicating weak positive correlations and suggesting minimal conceptual overlap between the HLSAC and GSE-5 instruments. [30]

**Table 1:** Comparison of measures by sex.

Measures	All			Female			Male			P-value
	N	Mean	SD	N	Mean	SD	N	Mean	SD	
HLSAC (10–40)	1410	30.12	5.17	713	29.82	5.14	697	30.43	5.19	0.026
GSE 5 (5-20)	1414	14.68	2.49	714	14.10	2.30	700	15.28	2.54	<0.001
KIDSCREEN Subscales										
Physical Well-Being	1421	46.53	9.94	715	43.99	8.52	706	49.18	9.66	<0.001
Psychological Well-being	1414	47.73	10.17	714	44.40	9.46	700	51.14	9.74	<0.001
Autonomy and Parent Relation	1417	50.94	9.30	715	49.56	9.26	702	52.33	9.12	<0.001
Peer and Social Support	1417	50.90	9.72	715	49.77	9.70	702	52.03	9.61	<0.001
School Environment	1413	48.04	9.39	714	47.13	9.47	699	48.98	9.22	<0.001

Note. HLSAC: Health Literacy of School-Aged Children measurement, GSE 5: General-Self-Efficacy Scale.

SD: Standard Deviation.

**Table 2:** Proportions of the proposed mediating effect of Self-Efficacy on the relationship between Health Literacy and HRQoL subscales by sex.

KIDSCREEN Subscales	S	N	ACME			P-value	ADE			P-value	Total effect			P-value	Proportion mediated			P-value
			Est.	95% CI			Est.	95% CI			Est.	95% CI			Est	95% CI		
Physical WB	F	713	0.69	0.44	0.94	<0.001	1.55	1.08	2.04	<0.001	2.24	1.80	2.04	<0.001	0.30	0.19	0.43	<0.001
	M	697	0.53	0.33	0.77	<0.001	0.96	0.53	1.43	<0.001	1.50	1.06	1.96	<0.001	0.36	0.22	0.55	<0.001
Psych. WB	F	713	0.97	0.73	1.22	<0.001	1.18	0.73	1.62	<0.001	2.15	1.72	2.58	<0.001	0.45	0.34	0.59	<0.001
	M	697	0.76	0.50	1.03	<0.001	0.44	−0.09	0.98	0.100	1.19	0.66	1.73	<0.001	0.63	0.39	1.14	<0.001
Aut./Parent Rel.	F	713	0.72	0.49	0.96	<0.001	1.05	0.54	1.56	<0.001	1.77	1.28	2.23	<0.001	0.41	0.27	0.61	<0.001
	M	697	0.45	0.25	0.70	<0.001	1.00	0.55	1.47	<0.001	1.46	1.03	1.89	<0.001	0.31	0.17	0.51	<0.001
Peer/Soc. Supp.	F	713	0.67	0.41	0.94	<0.001	0.68	0.20	1.16	0.008	1.35	0.93	1.77	<0.001	0.50	0.29	0.80	<0.001
	M	697	0.52	0.31	0.76	<0.001	0.17	−0.32	0.67	0.499	0.69	0.23	1.17	0.003	0.76	0.38	2.24	0.003
School Env.	F	713	0.89	0.65	1.15	<0.001	1.57	1.15	1.99	<0.001	2.47	2.08	2.85	<0.001	0.36	0.26	0.48	<0.001
	M	697	0.66	0.44	0.92	<0.001	1.20	0.77	1.64	<0.001	1.86	1.40	2.35	<0.001	0.36	0.24	0.50	<0.001

*Note.* Health Related Quality of Life: KIDSCREEN, Est = Estimate, S = sex, F = female, M = male, ACME = average causal mediation effect, ADE = average direct effect.The sum scores for health literacy and self-efficacy were transformed to a 0-100 scale. The T-scores for KIDSCREEN subscales were not transformed.

### 3.2 Mediation analysis

A hypothetical 10 percentage-point increase in the health literacy score in the proposed theoretical model (Fig. 2) resulted in significant increases across all HRQoL subscales (Table 2). The largest estimated total effect was observed for School Environment, with estimated increases in T-scores of 2.47 for girls and 1.86 for boys. These correspond to ≈ 1/4 SD for girls and ≈ 1/5 SD for boys.

Across all HRQoL subscales, the total effect estimates tended to be higher for girls than for boys. The estimated proportion of the mediated effect of self-efficacy was significant for all subscales, and ranged from 30% for Physical Well-Being for girls, to 76% for Peer and Social Support for boys (Table 2). Yet, for the subscale with the highest proportion mediated, the confidence interval was wide [i.e., Peer and Social Support among boys, (0.38-2.24)]

### 3.3 Sensitivity analysis

Sensitivity analyses indicated that the direct effect estimates (ADEs) were relatively robust to potential unmeasured confounding across all subscales in both sexes. An exception was observed for the Peer and Social Support subscale among boys (ρ ≥ −0.07), indicating greater sensitivity to unmeasured confounding, which was also reflected in the wide confidence intervals for the proportion mediated. The estimated mediated effects were otherwise relatively robust across subscales in both sexes (Table 3).

**Table 3:** Sensitivity analysis of direct and mediated effect estimates, by sex.

KIDSCREEN, subscales	ACME		ADE	
	ρ		ρ	
Sex	F	M	F	M
Physical Well-Being	≤0.25	≤0.24	≥-0.51	≥ −0.41
Psychological Well-Being	≤0.35	≤0.23	≥ −0.41	≥ −0.21
Autonomy and Parent Relations	≤0.26	≤0.21	≥ −0.36	≥ −0.43
Peer and Social Support	≤0.22	≤0.23	≥ −0.23	≥ −0.07
School Environment	≤0.33	≤0.32	≥ −0.53	≥ −0.52

*Note.* Health-Related Quality of Life: KIDSCREEN; F = female, M = male.

ACME = average causal mediation effect; ADE = average direct effect.

ρ (rho) represents the correlation between unmeasured confounders in the mediator–outcome relationship.

The values of ρ indicate the strength of unmeasured confounding required for the estimated effect to disappear or change direction.

## 4 Discussion

### 4.1 Main findings

In the present study, a significant association was found between health literacy and all HRQoL subscales. A hypothetical increase in health literacy was associated with higher HRQoL scores across all subscales. The estimated proportion mediated through self-efficacy was substantial for all subscales. The largest estimated total effects were observed in School Environment for both sexes. Overall, the proposed increase in health literacy tended to be somewhat larger for girls than for boys across all subscales.

Consistent with previous research findings, [1] suggesting that boys generally rate their overall health and well-being higher than girls, the boys in the present study scored significantly higher than girls in all subscales of HRQoL. On the other hand, compared with normative data, [24] girls in the current study reported substantially lower on Physical- and Psychological Well-Being. The narrow age range of participants in the current sample potentially introduces more homogeneity and may explain some of the deviation. HRQoL is found to decline during adolescence and the normative data included children from 8 to 18 years of age, while the current study mainly consisted of 13-year-olds. A recent Norwegian study focusing on 13–14 years old also reported lower levels of Physical and Psychological Well-Being for girls compared to the norm. [33] Another potential explanation for the low scores among girls in the present study is the timing of data collection. While the other studies collected data prior to the pandemic, the current study gathered information shortly after. The adverse health impacts of the lockdown, especially on girls, are well-documented. [34] The impact of the COVID-19 pandemic on health and well-being may persist over a long time. [35] Studies of the post-pandemic period are scarce, and future research is necessary to reveal possible long-term effects.

Based on a 13.5 percentage point variation in health literacy levels among adolescents across European countries, [9] the hypothetical 10 percentage point increase proposed in the present study seems realistic. The associations identified in this study suggest that higher health literacy is associated with more favorable health outcomes and are consistent with findings from a recent systematic review. [7] That review examined health literacy and health promotion among adolescents and found similar associations between health literacy and HRQoL across cross-sectional studies.

The observed mediation pattern is consistent with Bandura's social cognitive theory, which describes self-efficacy as a key determinant of how knowledge and skills are translated into behavior and coping. [36] From this perspective, health literacy may provide adolescents with the knowledge and competencies needed to understand and manage health-related situations, while self-efficacy may influence whether these competencies are translated into action. Adolescents who believe they can successfully apply their knowledge may therefore be better equipped to engage in health-promoting behaviors and cope with health-related challenges, which in turn may contribute to better HRQoL. Furthermore, self-efficacy is not only an individual characteristic but is also shaped by environmental influences, including mastery experiences, social support, and positive role models. [37] This theoretical perspective may help explain why adolescents with higher health literacy also report better HRQoL.

The largest estimated total effect of increasing health literacy was observed in the School Environment subscale for both sexes. The increase corresponds to approximately 1/4 of the standard deviation for girls and 1/5 for boys, which is noteworthy. From a public health perspective, the observed modest individual-level associations may have important implications at the population level. As proposed by Rose's population strategy, [37] interventions targeting determinants of health and well-being can generate substantial population-level benefits when applied across an entire population. [36] One possible explanation for the stronger association with the School Environment subscale is the conceptual overlap between health literacy and competencies that are relevant for learning. Students with higher health literacy may be better equipped to understand, evaluate, and use information, which could facilitate engagement with schoolwork and contribute to more positive perceptions of their school environment. [6] However, the confidence intervals around these estimates were relatively wide, indicating uncertainty regarding the size of the observed associations. Therefore, the apparently stronger association with the School Environment subscale should be interpreted cautiously and explored further.

One interpretation of the tendency towards larger estimated improvements in HRQoL among girls is that 13-year-old girls typically exhibit higher levels of emotional awareness and psychological distress and may be more receptive to health-related information [38,39] than boys of the same age. However, the confidence intervals for both the total and mediated effects overlapped between sex, suggesting that the tendency toward larger estimates among girls should be interpreted with caution. Nevertheless, the observed pattern generates interesting hypotheses for future research.

Previous studies have demonstrated that higher levels of self-efficacy positively influence academic performance, increase the likelihood of staying in school, and contribute to a better HRQoL. [20,40,41] The substantial proportion mediated through self-efficacy observed in the present analyses is consistent with theoretical models suggesting that improved health literacy can facilitate positive health outcomes, highlighting self-efficacy's role in effectively applying knowledge. [9,10]

### 4.2 Strengths and limitations

A major strength of this study is the relatively large population-based sample of 1425 participants, which enhances the reliability and generalizability of findings. Despite these strengths, there are some limitations to consider. The cross-sectional design limits the ability to evaluate the causal assumptions underlying the mediation analysis, particularly assumptions regarding temporal ordering. Therefore, our results should be regarded as exploratory. The relationships between the studied phenomena are complex, and although the proposed model is grounded in theory and previous research, it represents a simplified version of reality In addition, participants were recruited from 48 schools. School-level clustering resulted in a hierarchical data structure that was not accounted for in the mediation analyses. While this may have affected the precision of the estimates, clustering effects are often considered negligible when intra-class correlation coefficients (ICCs) are below 0.05, [32] suggesting that the impact on the present findings is likely to be limited.

Another limitation is that all measures are self-reported, potentially leading to various types of information bias. Participants may underreport or overreport outcomes due to social desirability or misinterpretation, despite the high internal consistency demonstrated for all instruments used.

Despite these limitations, the large population-based sample and the consistency of the findings across HRQoL subscales support the relevance of the study findings.

### 4.3 Implications

Assuming a causal relationship, increasing health literacy in the adolescent population may contribute to improved HRQoL. Strengthening health literacy within the population is emphasized in global health policy documents [42] as well as European health frameworks. [43] This framework, along with the Norwegian strategy to improve health literacy among citizens, [5,44] identifies the education sector as an important arena that includes the majority of children and adolescents from various socioeconomic backgrounds. Schools serve not only as institutions for academic development but also as a crucial environment for developing emotional and social competence. According to the WHO, Finnish children rank among those with the highest health literacy in Europe, attributed to the standardized framework of learning objectives for health literacy integrated throughout their school program, [45] which aims to ensure that every school-aged child acquires the skills necessary to manage their own health and the health of those around them.

A review of school-based health literacy programs emphasizes that the education- and health sectors must collaborate to improve health literacy. [46] While schools offer a sensible setting, teachers are not always confident in their knowledge about health. [47] School health services, located in schools, play a key role in public health work among adolescents in all Nordic countries, engaging in health promotion and prevention activities for all students. [48] In Norway, the mandate is outlined in the National Guideline for the School Health Services, [49] which strongly recommends individual health-promoting consultations with all 13-year-olds. These consultations aim to enhance students' health literacy, encourage positive health behaviors and self-efficacy, and identify students who may require follow-up. Additionally, the guideline underscores the importance of interprofessional collaboration with schools, including contributions to education on health topics. Despite the mandated role of the schools and the school health service, there is a lack of effectiveness studies focusing on how various strategies impact adolescents. Regular health literacy assessments in schools can possibly inform actions and effectively track improvement efforts. A study of school nursing in Germany [50] in which health literacy was measured among primary and secondary students before and 9-14 months after a school nurse provided primary care and health education based on the school's needs, showed that school health services and schools have a joint potential to enhance students' health literacy.

### 4.4 Conclusions

This study contributes to the literature by suggesting that hypothetical increases in health literacy may be associated with improvements in health-related quality of life and that general self-efficacy may be a potential mediator of this association. Future longitudinal studies are needed to examine the temporal ordering and causal nature of these relationships.

## Consent to participate

Participants were invited to join the study through information provided in the classroom by the school nurse and were asked if they wished to take part. In line with national regulations, participants below the age of 16 also needed parental informed consent to participate. All participants were informed of their right to withdraw from the study at any time. Confidentiality was maintained throughout the research process. This was ensured by using a de-identified ID in the data files with a linkage key stored separately. The researchers did not have access to the linkage key.

## Ethics statement

The Norwegian Regional Committees for Medical and Health Research Ethics [51] determined that the project falls under health service research, as its objective is not to generate new knowledge about health and disease and was therefore exempt from formal ethical approval. Nevertheless, the study was conducted in accordance with the ethical principles outlined in the Declaration of Helsinki. The study was assessed by the Norwegian Agency for Shared Services in Education and Research (Sikt) [52] (ref ID: 694124) to ensure compliance with ethical standards for data protection and information security. The GuideMe project was registered in ISRCTN prior to commencement (ID: 24173836).

## Consent for publication

Not applicable.

## Availability of data and materials

The datasets generated and analyzed during the current study are not publicly available due to the General Data Protection Regulation laws but will be provided by the corresponding author on reasonable request.

## Funding

The study is funded by the 10.13039/501100005416Norwegian Research Council (grant number 320097).

## Declaration of competing interest

The authors declare that they have no known competing financial interests or personal relationships that could have appeared to influence the work reported in this paper.
